# Geomechanical characterization of a heterogenous rock mass using geological and laboratory test results: a case study of the Niobec Mine, Quebec (Canada)

**DOI:** 10.1007/s42452-021-04617-1

**Published:** 2021-05-17

**Authors:** Shahriyar Heidarzadeh, Ali Saeidi, Catherine Lavoie, Alain Rouleau

**Affiliations:** Department of Applied Sciences, Université du Québec à Chicoutimi, Chicoutimi, QC G7H 2B1 Canada

**Keywords:** Rock mass characterization, Intact rock parameters, Lithological heterogeneity, Inherent variability, Laboratory tests, Niobec mine

## Abstract

**Abstract:**

To conduct a successful geomechanical characterization of rock masses, an appropriate interpretation of lithological heterogeneity should be attained by considering both the geological and geomechanical data. In order to clarify the reliability and applicability of geological surveys for rock mechanics purposes, a geomechanical characterization study is conducted on the heterogeneous rock mass of Niobec Mine (Quebec, Canada), by considering the characteristics of its various identified lithological units. The results of previous field and laboratory test campaigns were used to quantify the variability associated to intact rock geomechanical parameters for the different present lithological units. The interpretation of geomechanical similarities between the lithological units resulted in determination of three main rock units (carbonatite, syenite, and carbonatite-syenite units). Geomechanical parameters of these rock units and their associated variabilities are utilized for stochastic estimation of geomechanical parameters of the heterogeneous rock mass using the Monte Carlo Simulation method. A comparison is also made between the results of probabilistic and deterministic analyses to highlight the presence of intrinsic variability associated with the heterogeneous rock mass properties. The results indicated that, for the case of Niobec Mine, the carbonatite-syenite rock unit could be considered as a valid representative of the entire rock mass geology since it offers an appropriate geomechanical approximation of all the present lithological units at the mine site, in terms of both the magnitude and dispersion of the strength and deformability parameters.

**Article Highlights:**

- Evaluating the reliability and applicability of geological survey outcomes for rock mechanics purposes.
- A geomechanical characterization study is conducted on the heterogeneous rock mass by considering the various identified rock lithotypes.
- The geomechanical parameters of intact units and their associated variabilities are used to stochastically estimate the geomechanical parameters of the heterogeneous rock mass by employing the Monte Carlo Simulation.
- A comparison is also made between the results of probabilistic and deterministic geomechanical analyses.
- The results indicate that, in the case of Niobec Mine, the combined syenite-carbonatite rock unit could be considered as a valid representative of the entire rock mass.

## Introduction

Site characterization is an essential preliminary phase for implementing a successful rock mechanics program in any underground mining activity. As part of an underground mining plan, site characterization facilitates the subsequent geomechanical classifications by determining the geological settings and lithological characteristics of the area in which the mining activity is taking place. Aside from determining the geological settings and/or hydrogeological characteristics of the mining environment, site characterization contributes in estimation of the strength and deformability parameters of the numerous lithological units identified within the rock mass at the mine site [1, 2]. In fact, prior to any geomechanical investigation on rock mass, it is essential to characterize the intact rock in terms of lithological variability, by considering the mechanical properties of the key constituent lithological units [3–5].

From a geological perspective, various intact lithological units can be distinguished based on their lithological and petrographic differences. In some cases, numerous units could be identified within the rock having slight differences in mineral assemblage or alteration intensity [5, 6]. This approach of dealing with numerous identified lithological units may not be desirable in a geomechanical perspective since it could impose unnecessary complexities in rock mass classification/characterizations or numerical stability analysis. On the other hand, assuming only strong or only weak intact rock quality would either be too conservative or result in overestimation of rock mass strength [5, 7]. Therefore, a typical solution to deal with lithological heterogeneity of rock in a geomechanical perspective, could be through defining a periodic presence of strong and weak rock units with their corresponding geomechanical properties.

Lithological heterogeneity should be considered when estimating the geomechanical parameters of rock masses. Variable lithological compositions along with the presence of geological structures would result in anisotropy and heterogeneity in rock mass properties. Heterogeneity and anisotropy in rock mass geomechanical characteristics, originate from the presence of multiple rock formations having horizons with different alternations [2, 5]. The existing uncertainty in the strength and deformability parameters of a rock mass stems from the inherent variability associated with the rock formation process as well as a general lack of knowledge throughout the characterization process. The inevitable presence of such uncertainties in intact rock properties, complicates the process of rock mass characterization for a reliable estimate of rock mass properties [6, 8].

Different methods for estimating the strength and deformability parameters have been used in geomechanical characterization of rock masses. However, employment of the empirical Hoek–Brown failure criterion in conjunction with the Geological Strength Index (GSI) classification system has been reported to be the most common method for estimation of rock mass properties [9–11]. Conventional deterministic application of the above-mentioned approach, is not capable of addressing the intrinsic variable nature of rock masses since the inherent variability of the parameters are not taken into account. Probabilistic estimation of rock mass properties on the other hand, would be capable of incorporating the inherent variability of geomechanical parameters and depict a more realistic picture of the rock mass geomechanical behavior [6, 11–15].

The application of probabilistic methods in rock mass geomechanical characterization have been studied in many researches at different domains of geotechnical engineering such as rock slope stability e.g. [16–19] and underground rock mechanics such as tunnel stability [20–23], stope stability [24–26] and pillar stability [27–29]. However, few studies used probabilistic approaches to quantify the impact of lithological heterogeneity identified by geological surveys and laboratory tests on the uncertainties of the rock mass geomechanical characterization. As one of the pioneering studies, Kim and Gao [30] utilized the Monte Carlo Simulation (MCS) method to estimate the geomechanical parameters of a heterogeneous rock mass and demonstrated that the best-fitted statistical distribution for the mechanical properties of a basalt would be the third type asymptotic distribution of the smallest values. Hoek et al. [31] focused on characterization and determination of engineering properties of tectonically undisturbed sedimentary rock deposits (such as molassic formations) and recommended the use of empirical charts to estimate the GSI and *m*_*i*_ values of heterogeneous rock masses. Sari et al. [6], employed the MCS method, to incorporate the uncertainties associated with the intact rock strength and discontinuity parameters into a spread sheet model using statistical distributions, to quantify the variability of rock mass properties for Ankara andesite (Turkey). Tziallas et al. [4] determined the mechanical properties of heterogeneous rocks in the laboratory and proposed a methodology for predicting the rock mass strength of flysch formations consisting of siltstone–sandstone alternations in different proportions. Pepe et al. [5], used GSI classification system in conjunction with the Hoek–Brown failure criterion for geomechanical characterization of the highly heterogeneous Sanremo flysch formation located in Western Italy. Grenon et al. [32] evaluated two successive field and laboratory campaigns to investigate intact rock properties of an underground mining project in the Canadian arctic. The obtained results were compared to the pre-defined target levels of confidence associated with different stages of a mining project. More recently, Contreras et al. [33] applied the Bayesian methodology to estimate the intact rock strength parameters of the Hoek–Brown failure criterion, through the analysis of data from compression and tension tests; they explained the essential differences between frequentist and Bayesian statistics in quantifying the inevitable uncertainty in experimentally determined rock mechanics parameters.

Beside the accomplished researches, further studies are yet required to clarify how lithological and petrographic heterogeneities of a rock mass should be interpreted to provide a useful input for the geomechanical characterization. Identification of numerous lithological units within a rock mass (as the output of geological site characterizations) will not necessarily provide reliable data for rock mechanics experts since the geomechanical parameters of the identified lithological units may not show considerable differences. In fact, the best practice to deal with numerous identified lithological units within rocks should be developed and characterized for rock mechanics experts, not only to increase the efficiency but also to decrease the confusion caused by the presence of dozens of different lithological units, in geomechanical characterization process of heterogeneous rock masses.

To this end, this study uses the results of geological surveys to conduct a geomechanical characterization of rock, in order to evaluate the reliability and applicability of geological survey results for rock mechanics and rock engineering purposes. Geomechanical characterization is conducted on the intact rock specimens collected from the Niobec Mine (Quebec, Canada). Lithological and geomechanical properties of each identified lithological units at the mine, would be brought together from the results of previously conducted field and laboratory test campaigns. lithological similarities between the various constituent lithological units along with the expert judgment would be considered to identify main representative rock units at the Niobec Mine. Subsequently, the geomechanical parameters of each rock unit would be determined and their associated variabilities would be quantified and used to estimate the geomechanical parameters of the entire heterogeneous rock mass. For this purpose, MCS method will be employed to provide a stochastic estimation of strength and deformability parameters of rock mass using Hoek–Brown failure criterion in conjunction with GSI classification method. A comparison will also be made between the results of probabilistic and deterministic analyses to highlight the presence of intrinsic variabilities associated to the heterogeneous rock mass properties. Finally, a valid representative rock unit will be chosen over the others providing the most reasonable and reliable approximation of the geomechanical characteristics for the entire rock mass at the Niobec Mine.

## Niobec Mine

**Fig. 1:**
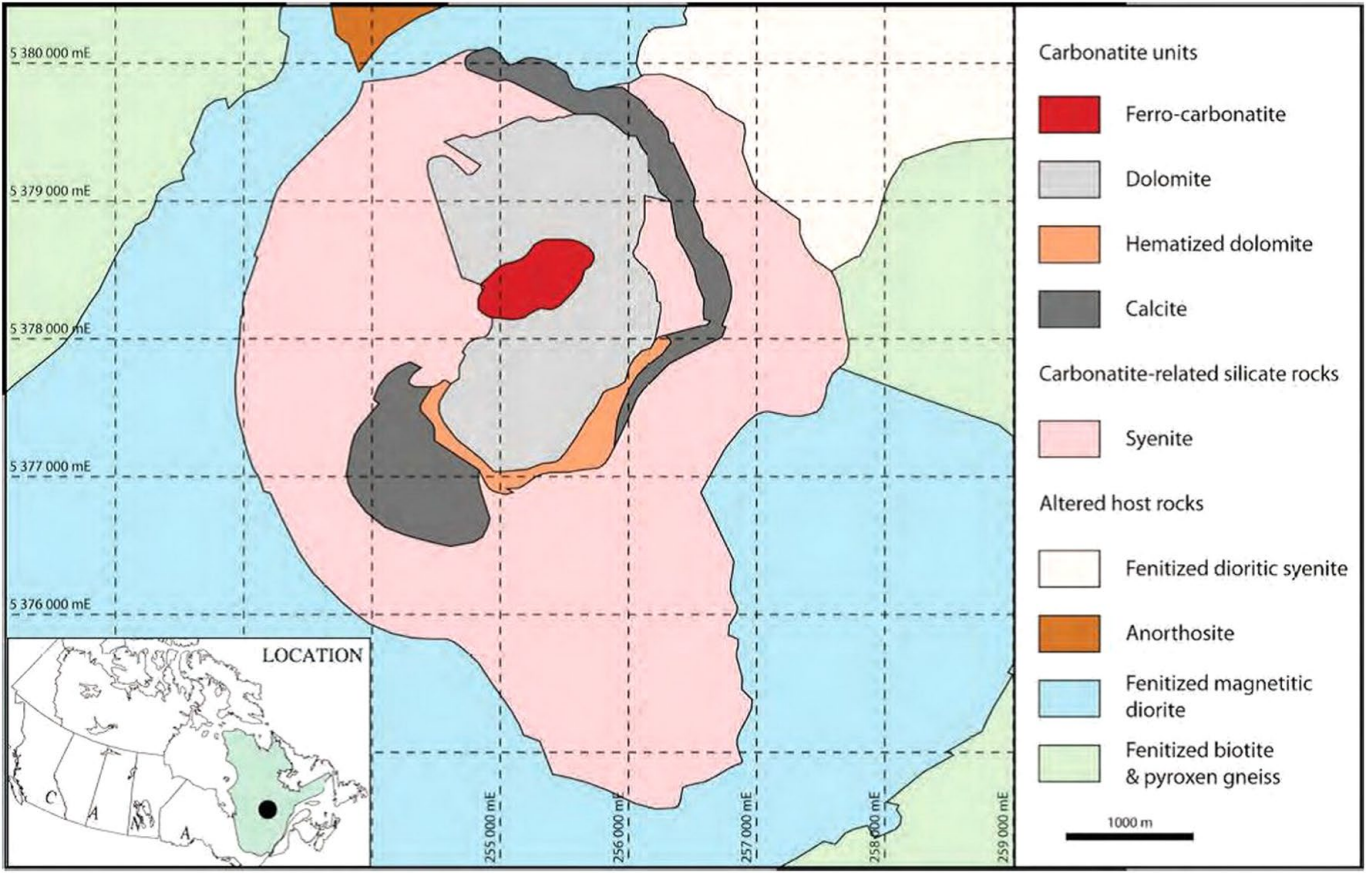
Geological map of the Saint-Honoré alkaline complex (after Tremblay et al. 2017)

The Niobec underground mine as part of the Saint-Honoré alkaline complex is located 13 km northwest of the city of Saguenay (Chicoutimi) within the limits of the municipality of Saint-Honoré, Quebec, Canada (Fig. 1).

The local geology of the region consists of an elliptical-shaped late Precambrian/early Cambrian intrusion core of alkaline silicates (syenite) and dolomitic/calcitic carbonatites [34]. The intrusion is overlain by an average of 75 m of Paleozoic limestone and dolomite of Trenton group [35]. Carbonatite in the intrusion core may have a fine to coarse grain dolomitic or calcitic composition. Carbonatite is brecciated or foliated in texture and contains variable amounts of accessory minerals (such as biotite, magnetite, apatite, pyrite, chlorite and pyrochlore). The foliation is often defined by the alignment of accessory minerals which are constituted from 1 to 10% of the total mineral assemblage [34]. The brecciated carbonatite contains 15% to 90% of syenite fragments from a few centimeters to approximately 10 m in size, showing total or partial alteration to chlorite. Furthermore, the hematitic alteration of carbonatite is also observed with variable intensities. The intrusion core is surrounded by massive or brecciated syenite having different degrees of alteration to chlorite [35]. The zones of economic mineralization in the deposit correspond to irregular shaped sub-vertical lenses oriented towards east–west direction with variable thickness of 73–171 m [34].

### Geological description of the rock mass

As mentioned above, the core of the intrusion at the Saint-Honoré alkaline complex is formed by syenite and carbonatite and the whole deposit is surrounded by syenite [34]. By means of the thin section analysis and macroscopic images, the identified lithological units at the Niobec Mine were grouped into three main rock units based on their mineral compositions and content of syenite fragments namely carbonatite, syenite and brecciated carbonatite-syenite. Carbonatite which is competent in term of quality, can contain various accessory minerals such as magnetite, biotite, apatite, chlorite and pyrite whose concentrations vary from 1 to 10%. These minerals are often present as foliation in carbonatite without affecting the resistance of the unit. Carbonatite can be massive to brecciated and can be altered to hematite in different degrees. Carbonatite is brecciated when it contains from 15 to 90% of syenite fragments. Partial or total alteration to chlorite can occur, resulting in resistance reduction in the brecciated carbonatite. The resistance of syenite varies depending on its degree of alteration [34, 35].

A lithological classification system has been developed based on the rock’s composition, grain size and level of accessory minerals to standardize the information regarding the geological description of drill core samples [37]. The various rocks forming the alkaline complex are codified according to their main mineral compositions and categorized into different lithological units. Accordingly, the first category was distinguished by dominance of carbonatite in its composition, while the second category was distinguished by the dominance of syenite. Furthermore, the third category included lithologies associated with particular cases or any other lithological units excluded from the first two categories. The codes which were used to designate the different lithological units, start with the letter "S" if the rock contains between 50 and 95% of syenite fragments and with the letter "C" if contains less than 5% of syenite fragments. Moreover, if the rock contains between 5 and 49% of syenite in its composition, the suffix "S" was added to the type of carbonatite. The rock was designated by the code "Si" or "Sa" if it contains more than 95% of syenite and respectively when not altered and altered in chlorite. Table 1 presents the geological codes established and used by the Niobec Mine authorities to describe the different lithological units identified within the rock mass. A detailed description of the codes attributed to each lithological unit utilized in Table 1, is provided by Lajoie [35].

**Table 1:** The codified classification established and used by the Niobec Mine to describe the different lithological units. Adapted from Golder [34] and Lavoie [37]

1	C1	13	C3CS	25	C3W	37	SC2	49	SCCA
2	C2	14	C3D	26	C5	38	SC3A	50	BR
3	C2C	15	C3DS	27	C5bio	39	SC3B	51	Bt
4	C2CS	16	C3N	28	C5S	40	SC3C	52	Cal
5	C2S	17	C3NA	29	C6	41	SC3N	53	LAMP
6	C2sucre	18	C3NAS	30	C6S	42	SC3NA	54	SM
7	C3A	19	C3NB	31	C9	43	SC3NB	55	Null
8	C3AS	20	C3NBS	32	C9S	44	SC3NC		
9	C3B	21	C3NS	33	CCA	45	SC3NT		
10	C3BS	22	C3NT	34	CCAS	46	SC5		
11	C3Bt	23	C3NTC	35	Si	47	SC5bio		
12	C3C	24	C3NTS	36	Sa	48	SC9		
C3C: Calcitic carbonatite containing a few accessory minerals									
C3N: Fine-grained dolomitic carbonatite containing a few accessory minerals. Colored as Dark wine red. Partially altered in hematite									
C3NA: Fine-grained dolomitic carbonatite containing variable concentrations of biotite and apatite. Normally foliated. Contains below 5% of magnetite									
C3NB: Fine-grained dolomitic carbonatite containing variable concentrations of biotite and apatite. Normally foliated. Contains over 5% of magnetite									
C5: Medium to coarse-grained dolomitic carbonatite with no accessory minerals									
C5bio: Medium to coarse-grained dolomitic carbonatite with over 10% biotite									
C3A: Medium-grained dolomitic carbonatite with variable concentrations of biotite and apatite. Typically foliated with magnetite concentrations below 5%									
C3B: Medium-grained dolomitic carbonatite with variable concentrations of biotite and apatite. Typically foliated with magnetite concentrations over 5%									
Sy: Unaltered syenite									
Sa: Syenite altered in chlorite between 10 and 100%. In the case of 100% alteration, the code CH is attributed									

First category with the carbonatite dominance (from 1 to 34), second category with the syenite dominance (from 35 to 49) and the third category containing all the remaining lithological units (from 50 to 55)

## The geomechanical parameters of the intact rock

Over the past years, various mapping and drilling campaigns have conducted laboratory tests on core samples extracted from different locations within the rock mass at the Niobec Mine [35, 38–44] to determine the physical and mechanical properties of various existing intact rock units. Table 2 presents the type and number of laboratory tests conducted by different campaigns at the Niobec Mine. Detailed information on the methodologies and specifications of each measuring campaign is provided by Lajoie [35].

**Table 2:** The type and number of laboratory tests conducted by different campaigns at the Niobec Mine

Test campaigns, (year)	Type and number of conducted laboratory tests				
	Brazilian test	Three-point bending test	Uniaxial compression test	Triaxial compression test	Test for elastic moduli of intact rock
Bétournay, 1986	22	10	24		34
Labrie 1987	155		79	14	12
Labrie 1997	25		6	12	18
Desbiens 1997	16	16	35	31	17
Corthésy 2000	17		17	6	
Labrie and Conlon 2005			5		5
Lajoie 2010	9	11	26	19	5
Grenon 2013	43		43		
Total	287	37	235	82	91

**Fig. 2:**
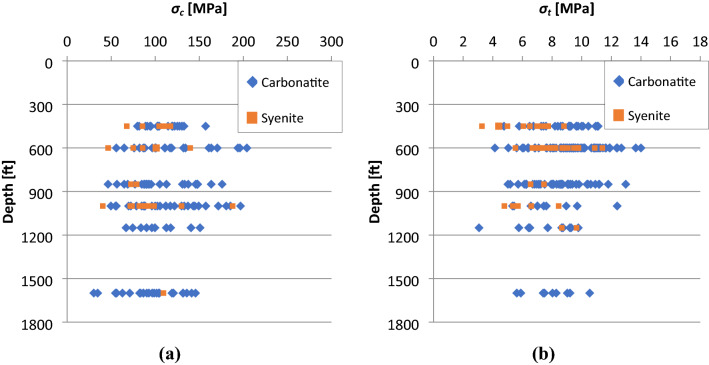
Distribution of the results as a function of depth for **a** uniaxial compressive strength **b** tensile strength

**Fig. 3:**
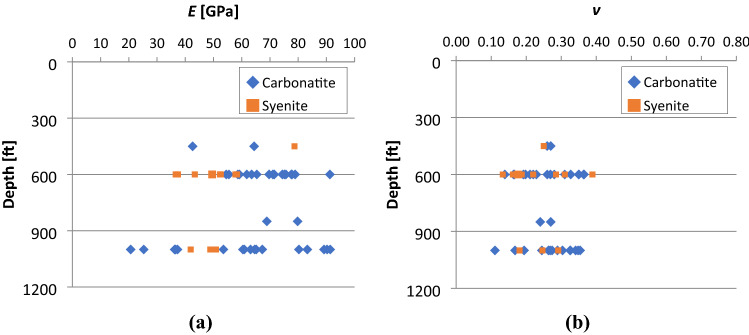
Distribution of the results as a function of depth for **a** Young’s Modulus and **b** Poisson’s ratio

The laboratory test results for all the campaigns were compiled and gathered into a database to facilitate the analysis of data. The generated dataset was also revised by Itasca [45] as part of a feasibility study conducted on the mine operation to assess the possibility of applying block caving method. The results were firstly grouped based on similarities of the identified lithological units and secondly based on the depth at which the samples were taken. The analysis of the results indicated that the variation of geomechanical properties, is mainly caused by the lithological differences rather than the depth. In fact, review of the laboratory test results demonstrated that the intact geomechanical properties show large variations in the average values calculated for different lithological units within the same test campaign (Tables 3 and 4). Considering the intrinsic nature of the rock materials, there is an uncertainty associated with each of the intact rock parameters, especially when compared to man-made materials. This uncertainty is generated through the process of formation and continuous modification of rock over its geologic history and results in both micro and macro scale property variations from one spatial location to another [8]. The variability associated with the intact rock’s strength and deformability parameters are presented in Figs. 2 and 3.

**Table 3:** The average values of Young’s modulus and Poisson’s ratio for each lithological unit

Test campaign, (year)	*E *_Ave._ [GPa]	*s.d* [GPa]	Number of tests	*ν*_Ave._ [MPa]	*s.d*	Number of tests
*Labrie (1987)*						
C5	77.40	2.23	4	0.30	0.04	4
C3A	55.75	18.60	2	0.26	0.02	2
C3C	58.15	5.16	2	0.31	0.06	2
C3N	64.40	–	1	0.26	–	1
Sy	37.00	0.57	2	0.26	0.05	3
*Labrie (1997)*						
C5	64.85	7.39	6	0.24	0.09	6
C3N	74.38	10.65	6	0.23	0.08	6
Sy intact	53.39	4.52	3	0.25	0.12	3
Sy altered	51.25	7.28	3	0.19	0.08	3
*Desbiens (1997)*						
C5	76.15	18.42	2	0.31	0.06	2
C5bio	90.25	–	1	0.29	–	1
C3A	59.66	5.64	3	0.19	0.08	3
C3N	84.95	5.74	3	0.32	0.01	3
C3NB	64.28	3.52	3	0.23	0.05	3
Sy altered	47.21	4.68	3	0.24	0.06	3
*Labrie and Conlon (2005)*						
C3B	36.66	0.51	3	0.34	0.00	2

*s.d  standard deviation

**Table 4:** The average uniaxial compressive and tensile strength values for each lithological unit

Test campaigns, (year)	*σ*_*c* Ave._ [MPa]	*s.d* [MPa]	Number of tests	*σ*_*t* Ave._ [MPa]	*s.d* [MPa]	Number of tests
*Labrie (1987)*						
C5	120.15	35.81	40	9.64	1.71	68
C3A	93.93	29.68	5	7.25	1.39	19
C3C	85.58	21.81	7	8.99	1.35	18
C3N	109.25	23.30	9	8.46	2.06	13
Sy intact	99.06	16.94	7	8.23	1.21	15
Sy altered	68.70	16.48	4	5.72	1.29	11
*Labrie (1997)*						
C5	107.19	35.23	2	7.04	2.02	9
C3N	147.99	68.04	2	10.25	1.42	7
Sy intact	139.71	–	1	8.97	0.64	5
Sy altered	85.75	–	1	8.96	1.63	4
*Desbiens (1997)*						
C5	126.47	32.31	6	8.66	1.48	2
C5S	121.89	–	1	–	–	–
C3A	106.15	72.41	2	–	–	–
C3B	117.89	13.69	4	7.76	1.69	2
C3N	118.19	38.10	5	6.07	1.19	3
C3NA	115.89	56.24	3	12.94	–	1
C3NB	163.10	29.37	3	7.97	1.35	2
Sy intact	87.92	10.63	4	5.69	1.28	2
Sy altered	83.30	28.93	6	6.50	1.70	3
*Corthésy (2000)*						
C5S	111.94	36.02	5	7.01	1.16	5
C3C	94.61	13.95	5	9.25	0.42	4
Sy	–	–	–	9.16	0.70	2
*Labrie and Conlon (2005)*						
C3B	66.45	13.85	5	–	–	–
*Lajoie (2010)*						
C5	116.21	27.78	2	–	–	–
C5bio	97.74	6.86	3	–	–	–
C3A	89.03	36.08	9	8.59	1.72	5
C3AS	67.38	35.23	3	–	–	–
C3B	104.91	30.47	7	7.14	1.05	4
SC5	109.30	–	1	–	–	–
*Grenon (2013)*						
C5	141.63	–	1	11.22	3.73	2
C3A	134.94	31.13	11	11.06	1.10	11
C3B	134.27	20.98	6	10.22	2.83	8
C3C	108.12	29.18	5	9.25	1.65	6
C3A/C3B	168.04	12.03	2	13.20	0.09	2
SC3C	114.17	–	1	–	–	–

*s.d  standard deviation

Inherent variability in uniaxial compressive strength (*σ*_*c*_) and tensile strength (*σ*_*t*_) stems from the variability of index properties and petrographic characteristics of rock. According to Langford and Diederichs [8], index properties (e.g., the total porosity, density, water content and durability) should be consistent for the rock specimens taken from the same formation (lithological context). While, variations in petrographic characteristics develop different patterns of micro cracks within each rock sample, resulting in variability of uniaxial compressive and tensile strength values. Variability in petrographic characteristics is defined by the variation in grain size and shape, texture (degree of interlocking), micro fractures (grain boundaries, mineral cleavages), nature of cement, degree of chemical alteration and anisotropy (orientation of the cracks and the condition of stress distribution). Similarly, variability in the deformability parameters such as Young’s Modulus and the Poisson’s ratio is generated due to the variation in parameters such as porosity and degree of jointing, water content and the vibration effects from blasting [8].

In order to quantify the existing variability associated to the intact strength and deformability parameters at the Niobec Mine, lithological units were examined in different categories. Firstly, the strength and deformability parameters of the carbonatite units containing few accessory minerals (C5, C3N and CCA) were compared with those of the foliated units and containing many accessory minerals (C3C, C3A, C3NA, C3B and C3D). Secondly, the strength and deformability parameters of calcific units (C3C, C3D and CCA), were compared to those of dolomitic (C3A, C3B, C5, C3N and C3NA) and syenitic units. Finally, third comparison was made between the strengths and deformability parameters of northern units (C3N, C3NA and C3NT) and all other units [37]. The average values of Young’s modulus and Poisson’s ratio as well as the uniaxial compressive and tensile strength for each lithological unit obtained from the previous test campaigns are presented respectively in Tables 3 and 4.

Studying the obtained results specified that the largest variations belong to the carbonatitic lithological units. These variations in strength and deformability parameters showed a significant dependence on the degree of alteration.

**Fig. 4:**
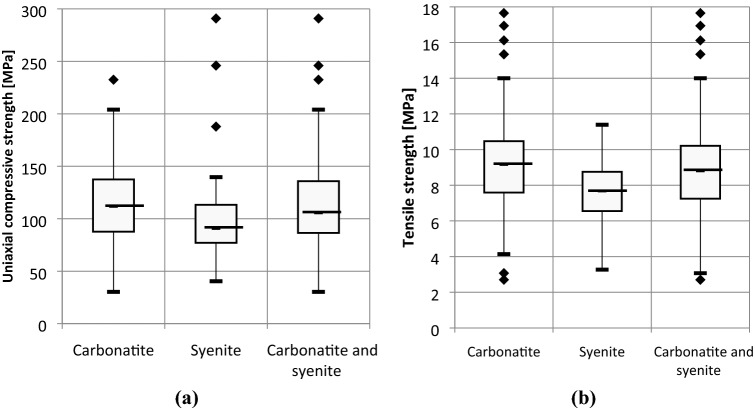
Box-plot diagrams for the results of **a** uniaxial compressive and **b** tensile strength of syenite, carbonatite and carbonatite-syenite units

**Fig. 5:**
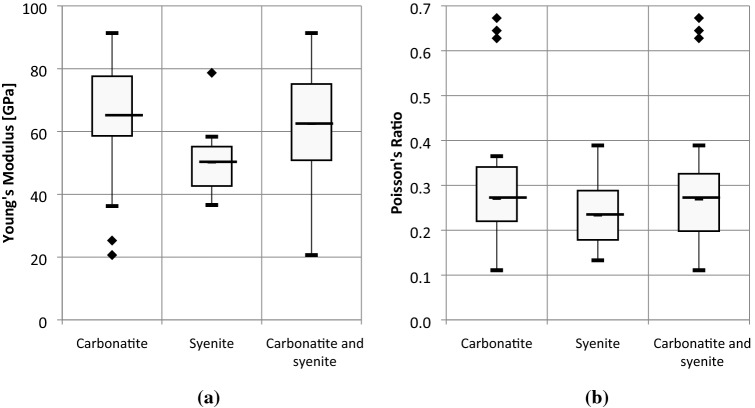
Box-plot diagrams for the results of **a** Young’s Modulus and **b** Poisson’s Ratio of syenite, carbonatite and carbonatite-syenite units

Box-plot diagrams (Figs. 4 and 5) illustrate the dispersion of strength and deformability test results. Box-plot diagrams are able to compare the series of obtained results for the main lithological units and identify the outliers to be excluded for further calculations. It was indicated that the syenite has lower quality than carbonatite in terms of geomechanical properties. The average values of strength and deformability parameters of carbonatite and syenite rock units were estimated by compiling all the data for carbonatitic and syenitic lithological units as presented in Tables 5 and 6 respectively. The number of tests considered in the calculations of the average values of strength and deformability parameters are shown in each table.

**Table 5:** The mean and standard deviation values of uniaxial compressive and tensile strength results for syenite, carbonatite and carbonatite-syenite units

Rock unit	Uniaxial compressive strength			Tensile strength		
	*σ*_*c* Ave._ [MPa]	*s.d* [MPa]	Number of tests	*σ*_*t* Ave._ [MPa]	*s.d* [MPa]	Number of tests
Carbonatite	113.06	35.72	153	9.05	2.06	191
Syenite	90.74	23.42	25	7.53	1.79	42
Carbonatite and syenite	110.36	35.46	179	8.75	2.12	234

*s.d  standard deviation

**Table 6:** The mean and standard deviation values of Young’s modulus and Poisson’s ratio results for syenite, carbonatite and carbonatite-syenite units

Rock unit	Young’s modulus			Poisson’s ratio		
	*E*_Ave._ [GPa]	*s.d* [GPa]	Number of tests	*ν*_Ave_	*s.d*	Number of tests
Carbonatite	67.12	14.68	36	0.26	0.07	35
Syenite	48.14	7.44	11	0.24	0.07	12
Carbonatite and syenite	61.41	17.19	50	0.26	0.07	47

*s.d  standard deviation

The parameters of Mohr–Coulomb and Hoek–Brown failure criteria for the intact rock were also determined from the results of the uniaxial, and triaxial compression tests carried out by the previous campaigns. The experimental results were first grouped together in order to determine the parameters such as *c*, *φ*, *m*_*i*_ and *σ*_*ci*_ for each of the present lithological units (Table 7). Accordingly, linear regression lines were fitted to the graphs generated from the results of the laboratory tests. The failure envelope curves were subsequently plotted for each of the identified lithological units.

The intact rock failure parameters such as the material constant *m*_*i*_ are the parameters mostly defined by the rock type. Variability of such parameters is normally caused by variability in mineral content (lithological heterogeneity), the degree of “particle interlocking”, foliation and grain size (texture) [8, 46]. According to Table 6, the intact failure parameter values of different carbonatite units, are relatively constant except for the units either with high alteration degrees or with high presence of accessory minerals. Moreover, based on the results of triaxial tests, intact specimens with a more uniform lithological context and less concentration of micro cracks, showed higher strength than the specimens with high concentration of accessory minerals, or with a large amount of syenite fragments altered in chlorite.

**Fig. 6:**
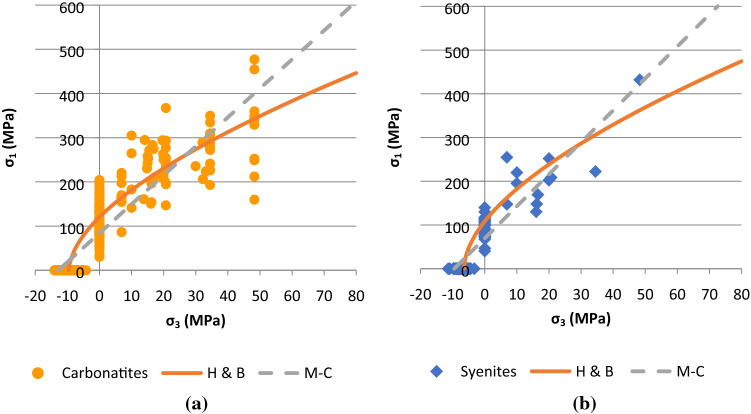
Hoek–Brown and Mohr–Coulomb failure envelope for **a** Carbonatite and **b** Syenite

**Fig. 7:**
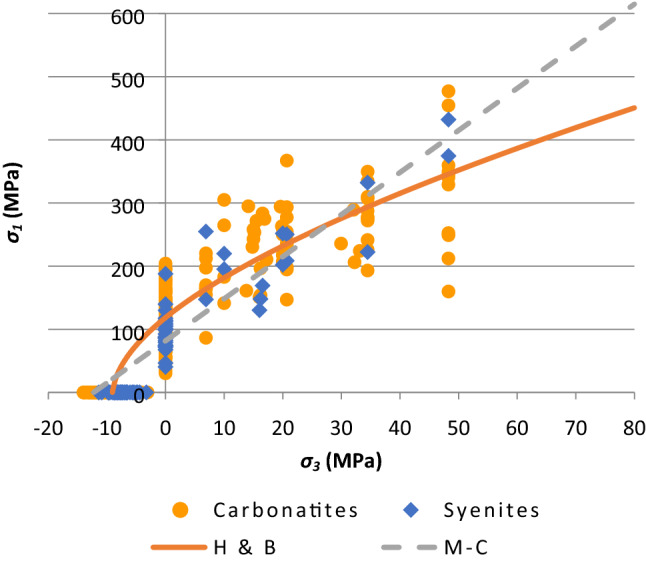
The combined Hoek–Brown and Mohr–Coulomb failure envelopes for the carbonatite and the syenite

The test results are then combined in order to compare the failure parameters of the carbonatite and syenite units. Figure 6 presents the obtained Mohr–Coulomb and Hoek–Brown failure envelopes for the groups of carbonatites and syenite rock units while Fig. 7 illustrates the obtained Mohr–Coulomb and Hoek–Brown failure envelopes for the carbonatite-syenite rock unit. The average values of the failure parameters for the three mentioned rock units are reported in Table 8.

According to the presented results in Table 7, the estimated values of failure parameters for carbonatite is slightly higher than syenite, but fairly close to the values obtained for the carbonatite-syenite rock unit.

Even though a slight difference was observed between the calculated strength and deformability parameters of carbonatite and syenite intact units, the carbonatite-syenite provided a quit fair approximation of the two units. Therefore, assuming a homogeneous intact rock containing a random combination of syenite and carbonatite units could provide an agreeable geomechanical description of intact rock for the case of Niobec Mine. Table 9 summarizes the average values to be considered in characterizing the properties of the intact rock at the Niobec mine.

**Table 7:** The parameters of Hoek–Brown and Mohr–Coulomb failure criteria for each lithological unit

Test campaigns (year) and lithological units	Failure criteria					
	Mohr–Coulomb			Hoek–Brown		
	*c* [MPa]	*φ* [°]	R^2^	*m*_*i*_	*σ*_*ci*_ [MPa]	R^2^
*Labrie (1987)*						
C5	16.72	56.56	0.8792	15.24	139.53	0.7747
C3A	12.12	55.03	0.9259	16.50	115.78	0.8722
C3C	12.68	47.71	0.8794	8.89	83.69	0.8746
C3N	14.90	56.00	0.8834	14.79	120.48	0.8185
Sy intact	13.21	51.86	0.8549	10.71	93.75	0.8906
Sy altered	8.67	47.24	0.8818	10.62	64.92	0.9224
*Labrie (1997)*						
C5	12.45	53.03	0.8488	14.51	115.28	0.7234
C3N	17.33	53.29	0.8401	13.46	148.59	0.6834
Sy intact	14.51	51.02	0.8939	11.50	115.80	0.9101
Sy altered	14.30	53.77	0.963	15.03	129.18	0.9437
*Desbiens (1997)*						
C5	17.71	48.42	0.6794	32.49	87.62	0.8581
C3A	30.85	29.66	0.7218	4.64	117.86	0.663
C3B	18.95	45.50	0.7269	17.35	108.44	0.8198
C3N	16.93	46.22	0.742	12.29	107.93	0.8171
C3NA	33.16	30.43	0.435	3.76	139.72	0.1985
C3NB	25.47	46.23	0.8138	14.48	150.62	0.7163
Sy intact	16.26	47.29	0.8118	19.76	108.95	0.7377
Sy altered	13.97	48.54	0.943	21.33	94.30	0.9631
*Corthésy (2000)*						
C5S	15.46	45.86	0.7835	10.93	107.40	0.7487
C3C	15.82	46.91	0.8573	12.71	112.10	0.71
*Lajoie (2010)*						
C5	35.15	27.03	0.8549	4.49	113.68	0.71
C3A	16.42	46.59	0.8668	14.39	109.39	0.93
C3AS	21.07	34.18	0.7351	6.73	91.01	0.5693
C3B	17.80	44.02	0.8926	13.16	107.37	0.9376

*R^*2*^ = *Regression coefficient*

**Table 8:** The values of Hoek–Brown and Mohr–Coulomb failure parameters

Rock unit	Failure criteria					
	Mohr–Coulomb			Hoek–Brown		
	*c* [MPa]	*φ* [°]	R^2^	*m*_*i*_	*σ*_*ci*_ [MPa]	R^2^
Carbonatite	16.40	47.32	0.7764	12.45	120.25	0.6845
Syenite	12.88	49.41	0.8820	16.60	108.42	0.8139
Carbonatite and syenite	15.82	47.65	0.7872	13.01	118.53	0.7012

*R^*2*^ = *Regression coefficient*

**Table 9:** The mean values geomechanical parameters for the carbonatite-syenite intact rock unit

Geomechanical properties	carbonatite-syenite unit
Uniaxial compressive strength	*σ*_*c*(mean)_ = 110.4 MPa
Tensile strength	*σ*_*t*(mean)_ = 8.8 MPa
Young’s modulus	*E*_(mean)_ = 61.4 GPa
Poisson’s ratio	*υ*_(mean)_ = 0.26
Cohesion (Mohr–Coulomb)	*c* = 15.8 MPa
Friction angle (Mohr–Coulomb)	*φ* = 47.7°
*σ*_*ci*_ (Hoek & Brown)	*σ*_*ci*_ = 118.5 MPa
*m*_*i*_ (Hoek & Brown)	*m*_*i*_ = 13.0
Unit weight of Rock	*γ* = 2.839 t/m^3^

## Geomechanical characterization of the rock mass

It was shown in the previous section that in the scale of intact rock, a homogenous carbonatite-syenite rock rather than considering only a carbonatitic or syenitic intact rock provides a more realistic approximation for the existing rock at the Niobec Mine. This assumption is aligned with the findings of local site characterization programs conducted during the deposit exploitation which emphasized that the nature of rock mass at the Niobec Mine is too complex to be discretized geomechanically hence the observed irregularities in the quality of intact rock units should be considered to occur in local scales [34, 35]. In this part, the same hypothesis is examined in the rock mass scale to see whether the results would support the consideration of a unified carbonatite-syenite rock mass unit for the Niobec Mine or not. Geomechanical characterization of rock mass, can be accomplished through using the obtained results of intact geomechanical parameters. MCS method is employed to provide a stochastic estimation of rock mass geomechanical parameters by incorporating the associated variabilities of intact parameters.

### Probabilistic estimation of rock mass geomechanical parameters

Hoek–Brown failure criterion is used in conjunction with the Geological Strength Index (GSI) to estimate the geomechanical parameters of the syenitic, carbonatitic and carbonatite-syenite rock mass. Accordingly, mean and standard deviation values of the intact UCS, the Hoek–Brown material constant (*m*_*i*_) and the intact Young’s modulus (*E*_*i*_) of syenite, carbonatite and the carbonatite-syenite rock units are adopted from the intact rock characterization results (Table 10). The mean GSI value and the corresponded standard deviation of the rock mass were adopted from the study conducted by Lavoie [37]. Based on the concentration of discontinuities and the number of joint sets identified within the carbonatite-syenite unit, the rock mass was recognized to have a blocky structure with rough and slightly altered joint surface condition. Therefore, to assign an appropriate value of GSI to the rock mass at the Niobec Mine, the calculated value of RMR_89_ as equal to 71 was used (as reported by Golder [34] and Lajoie [35]) and the GSI was calculated as 74 based on the conversion formula provided by Hoek and Brown [3]. It should be noted that estimating the GSI value from RMR_89_ might be unreliable, especially for poor quality rock masses and for rocks with lithological peculiarities that cannot be easily incorporated in the RMR classification [9]. However, in this case, due to the high quality of rock mass and lack of GSI data resulted from direct GSI estimations, the above-mentioned conversion formula is used.

**Table 10:** The mean and standard deviation values of geomechanical parameters for the syenite, carbonatite and carbonatite-syenite rock

	carbonatite-syenite		Syenite		Carbonatite	
	Min–Max	Mean–S.d	Min–Max	Mean–S.d	Min–Max	Mean–S.d
*UCS*_intact_	30.3–204	110.4–35.5	40.5–139.7	90.7–23.4	30.3–204	113.1–35.7
GSI	36–100	74.3–8.2	36–100	74.3–8.2	36–100	74.3–8.2
*m*_*i*_	4.5–22	13.0–4.3	10.5–22	16.6–4.7	4.5–24	12.4–4.1
*E*_*i*_	20.7–91.4	61.4–17.2	36.6–58.3	48.1–7.4	36.2–91.4	67.1–14.7
*υ*	0.11–0.39	0.26–0.07	0.13–0.39	0.24–0.07	0.11–0.73	0.26–0.07

Since the reported RMR_89_ value was estimated for the entire rock mass and no GSI value was available separately for different units within the rock mass, the same GSI value of 74 was considered for each syenite and carbonatite units for further geomechanical calculations (Table 10). These values are considered as random inputs when calculating the geomechanical parameters.

The Kolmogorov – Smirnov statistical test was applied on the above-mentioned parameters to assign the best-fitted probability distributions function (PDF) to each parameter. According to the results of Akaike Information Criterion (AIC) goodness of fit test, Weibull and Normal distributions were determined to be the most appropriate statistical distributions for the parameters. However, for the sake of simplicity, normal PDFs were chosen to be assigned to the parameters. Assuming normal distribution function to describe the random characteristics of the geomechanical parameters is consistent with the suggestions made by many studies e.g. [6, 20, 22, 26, and 47].

**Fig. 8:**
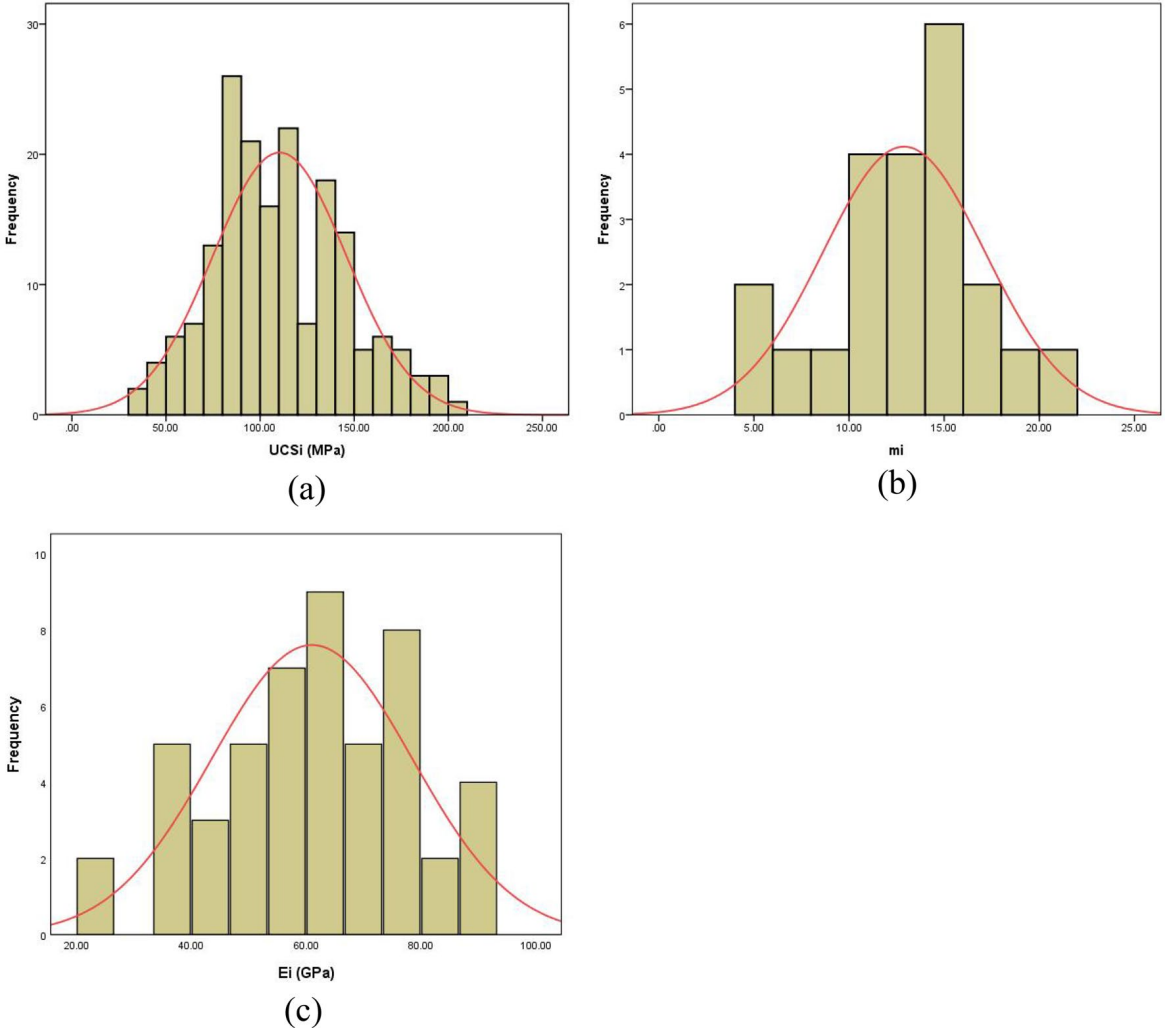
The truncated normal probability distribution functions (PDFs) for **a** the uniaxial compressive strength, **b** the Hoek–Brown material constant m_i_, and **c** Young’s modulus E_i_ of the carbonatite-syenite intact rock

To eliminate generation of negative and/or false values, the normal PDFs were truncated by using the actual reported minimum and maximum values. Figures 8 a–c illustrate examples of the truncated PDF plots for intact rock material properties of the carbonatite-syenite rock mass.

Subsequently, the MCS method was applied to calculate the Hoek–Brown strength and deformability parameters of each rock mass type through Eqs. 1–6 [9].

1 $$m_{b} = m_{i} {\text{e}}^{{(\frac{GSI - 100}{{28 - 14D}})}}$$

2 $$s = {\text{e}}^{{(\frac{GSI - 100}{{9 - 3D}})}}$$

3 $$a = \frac{1}{2} + \frac{1}{6}(e^{{\frac{ - GSI}{{15}}}} - e^{{\frac{ - 20}{3}}} )$$

4 $$\sigma_{cm} = \sigma_{ci} s^{a}$$

5 $$\sigma_{t} = \frac{{s\sigma_{ci} }}{{m_{b} }}$$

6 $$E_{rm} = E_{i}\left (0.02 + \frac{{1 - \frac{D}{2}}}{{1 + e^{{(\frac{60 + 15D - GSI}{{11}})}} }}\right)$$

where *D* represents the degree of disturbance of the rock mass (ranging from 0 to 1 for undisturbed in-situ rock masses to highly disturbed rock masses) [9]. Since the rock mass is assumed to be undisturbed by controlled blasting, the parameter *D* is considered equal to 0 for this study.

Each output parameter was generated from 10,000 iterations of randomly selected combinations of input parameters in accordance with their assigned PDF using Latin hypercube sampling algorithm (LHS). The advantage of LHS is that it provides smoother resulting PDFs with fewer iterations by using stratified sampling models. For the sake of simplicity, all the input parameters were assumed as independent variables. Although the dependence of the Hoek–Brown parameters could jeopardize the probabilistic estimation of the output parameters, Sari et al., [48] stated that the dependence between the Hoek–Brown parameters does not have a significant effect on the estimation of strength and deformability parameters using probabilistic simulations.

By the aim of MCS, the variability associated to the output geomechanical parameters were quantified and the Kolmogorov–Smirnov test determined the best-fitted distribution function for each parameter. The mean, standard deviation and best-fitted PDF of strength and deformability parameters for each rock mass type are reported in Table 11.

**Table 11:** The mean values and standard deviations of geomechanical parameters for the syenite, carbonatite and carbonatite-syenite rock mass

PDF		Carbonatite-syenite				Syenite				Carbonatite			
		Mean	*S.d*	Min	Max	Mean	*S.d*	Min	Max	Mean	*S.d*	Min	Max
*m*_*b*_	Gamma	5.45	2.32	0.88	19.2	6.83	2.41	1.69	19.17	5.28	2.25	0.925	18.56
*s*	Log-normal	0.08	0.07	0.00	0.97	0.057	0.092	0.00	0.979	0.086	0.09	0.001	0.99
*a*	Log-normal	0.501	0.0008	0.500	0.508	0.501	0.0008	0.500	0.508	0.501	0.0008	0.500	0.509
*σ*_*c*_	Inversed-gaussian	29.5	17.20	2.33	185.2	24.06	13.03	2.46	114.56	30.09	17.36	1.84	162.9
*σ*_*t*_	Log-normal	1.64	1.44	0.05	22.6	1.000	0.76	0.045	8.175	1.73	1.50	0.049	15.89
*E*_*rm*_	Normal	47.2	14.25	8.70	88.3	37.44	7.48	9.61	56.81	51.82	12.98	10.34	88.89

**Fig. 9:**
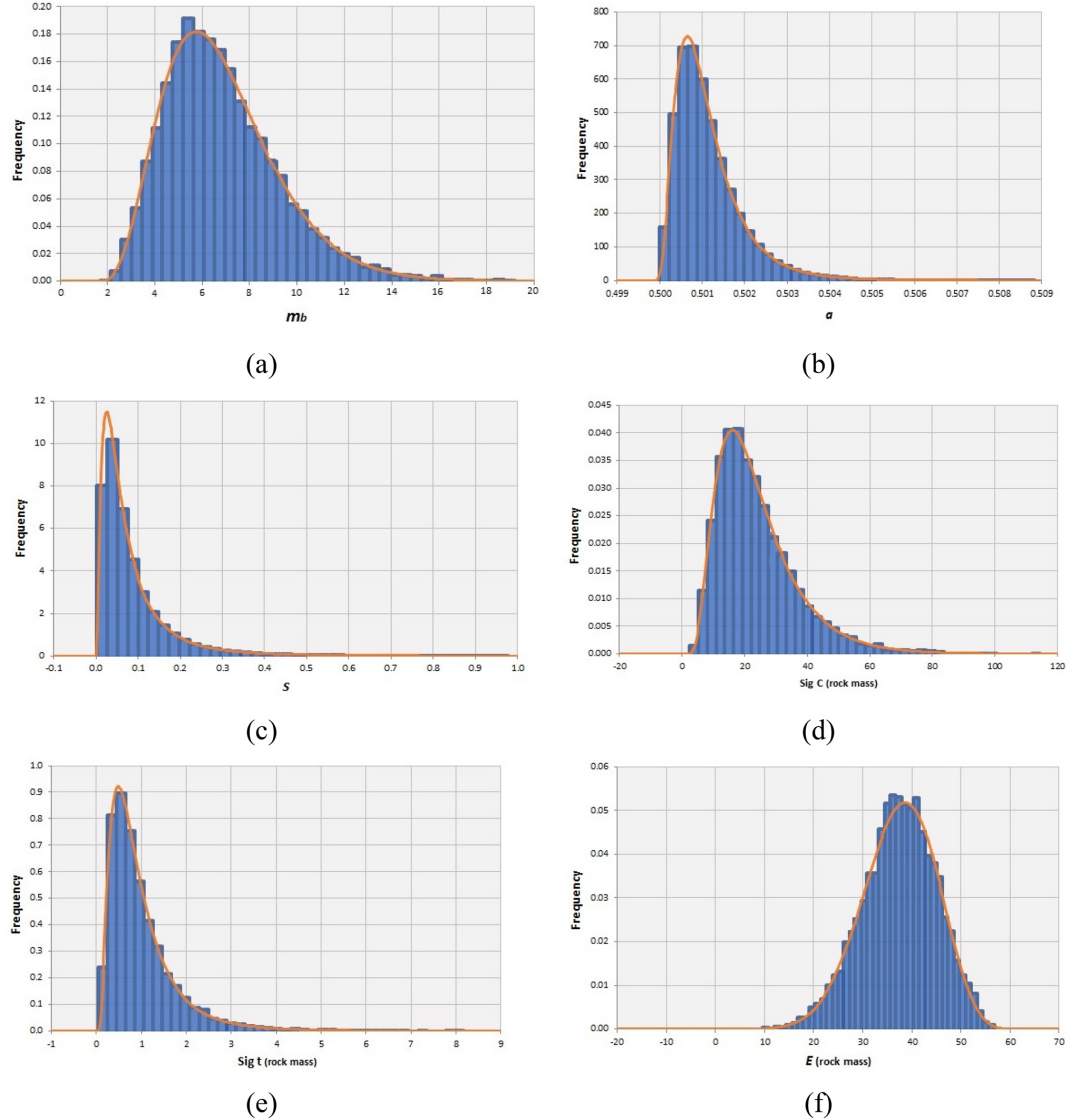
Histograms and the best-fitted PDF of the geomechanical parameters of the syenite rock mass

**Fig. 10:**
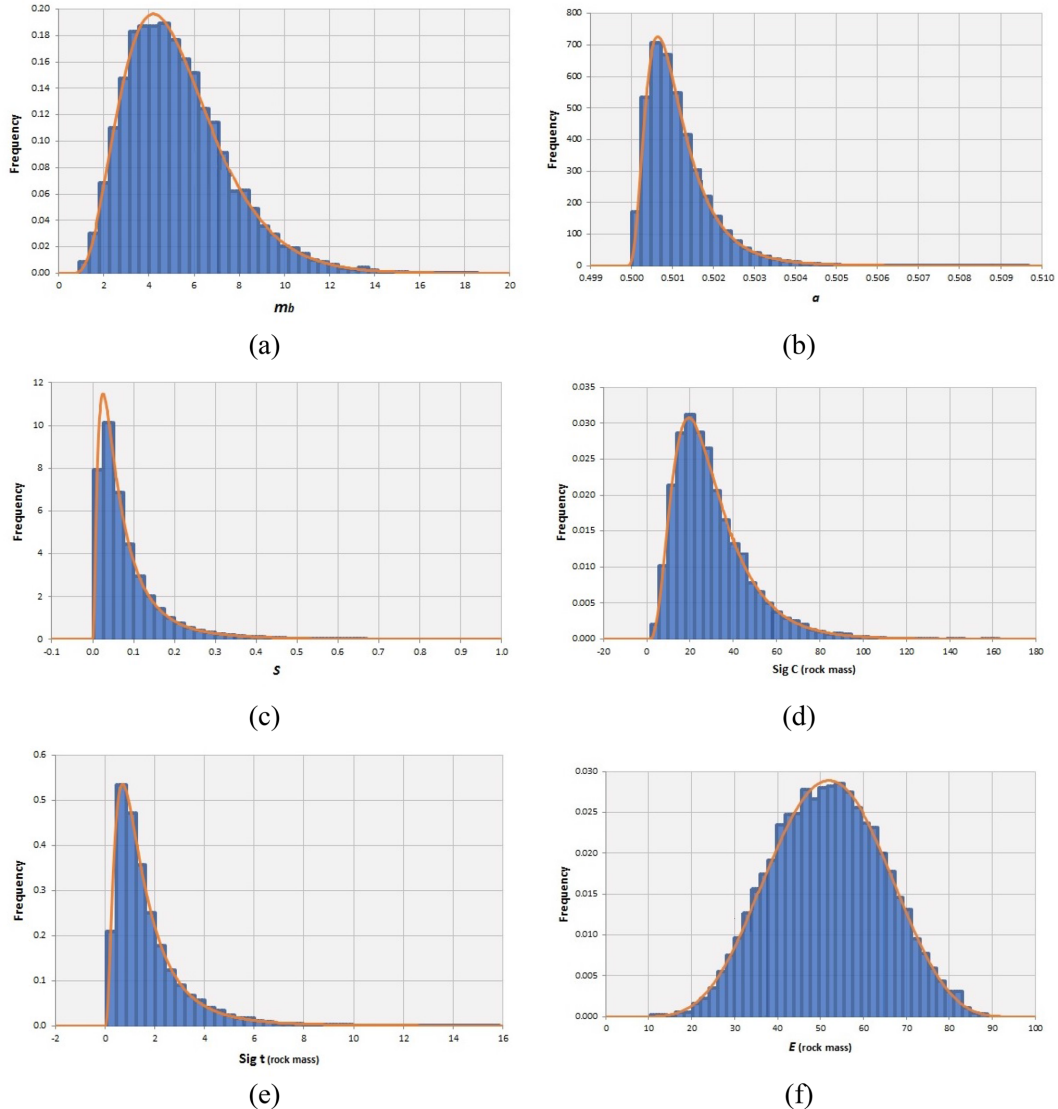
Histograms and the best-fitted PDF of the geomechanical parameters of the carbonatite rock mass

**Fig. 11:**
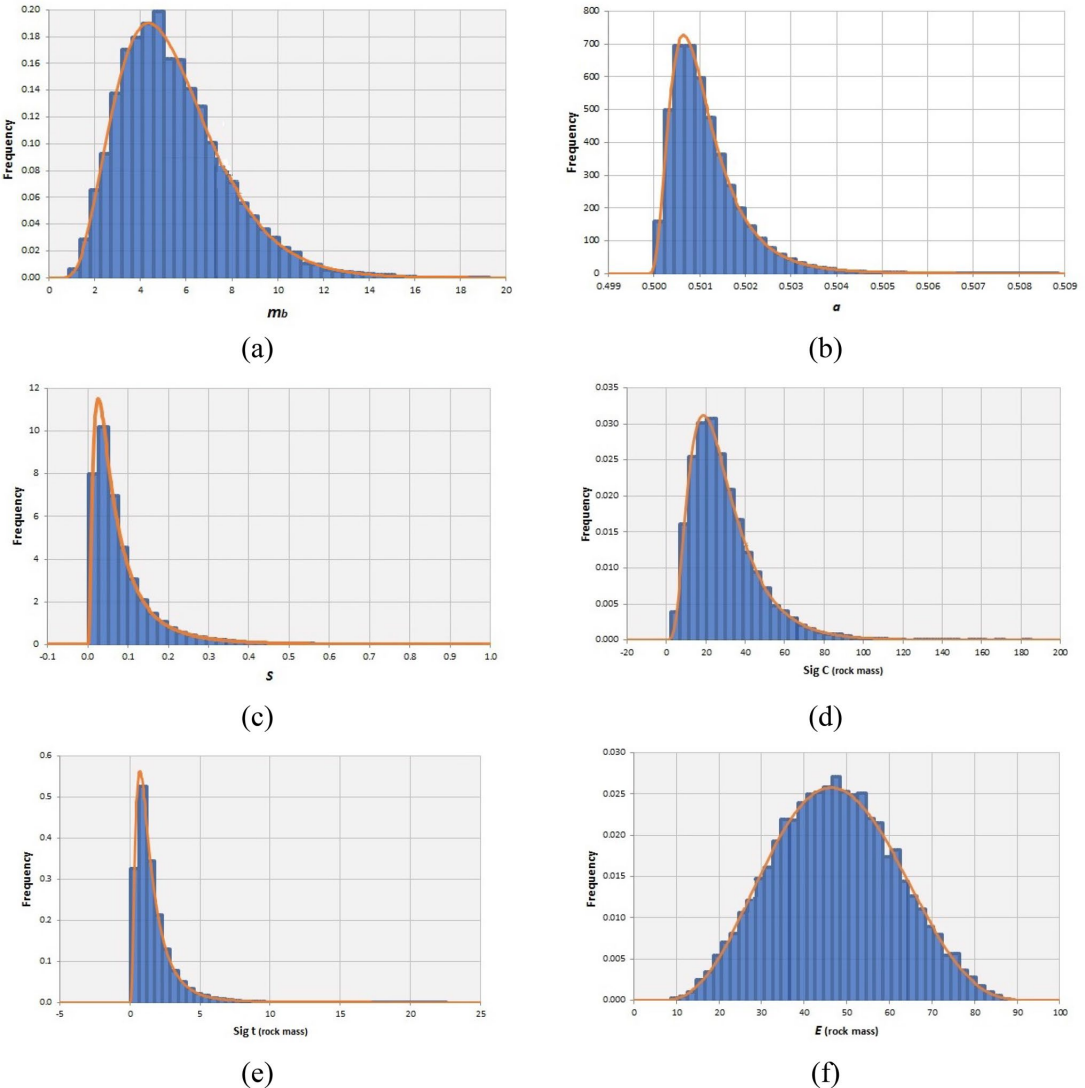
Histograms and the best-fitted PDF of the geomechanical parameters of the carbonatite-syenite rock mass

Figures 9, 10, 11a–f respectively illustrate the obtained truncated PDF plots of output geomechanical parameters for syenite, carbonatite and carbonatite-syenite rock masses.

The results of rock mass geomechanical characterization indicated that, similar to the intact properties, the obtained values for syenite and carbonatite rock masses show slight differences and the carbonatite rock mass quality was estimated to be higher than syenite. However, estimated geomechanical parameters for the carbonatite-syenite rock mass were fairly close to the values calculated only for the carbonatite.

### Deterministic estimation of rock mass geomechanical parameters

**Fig. 12:**
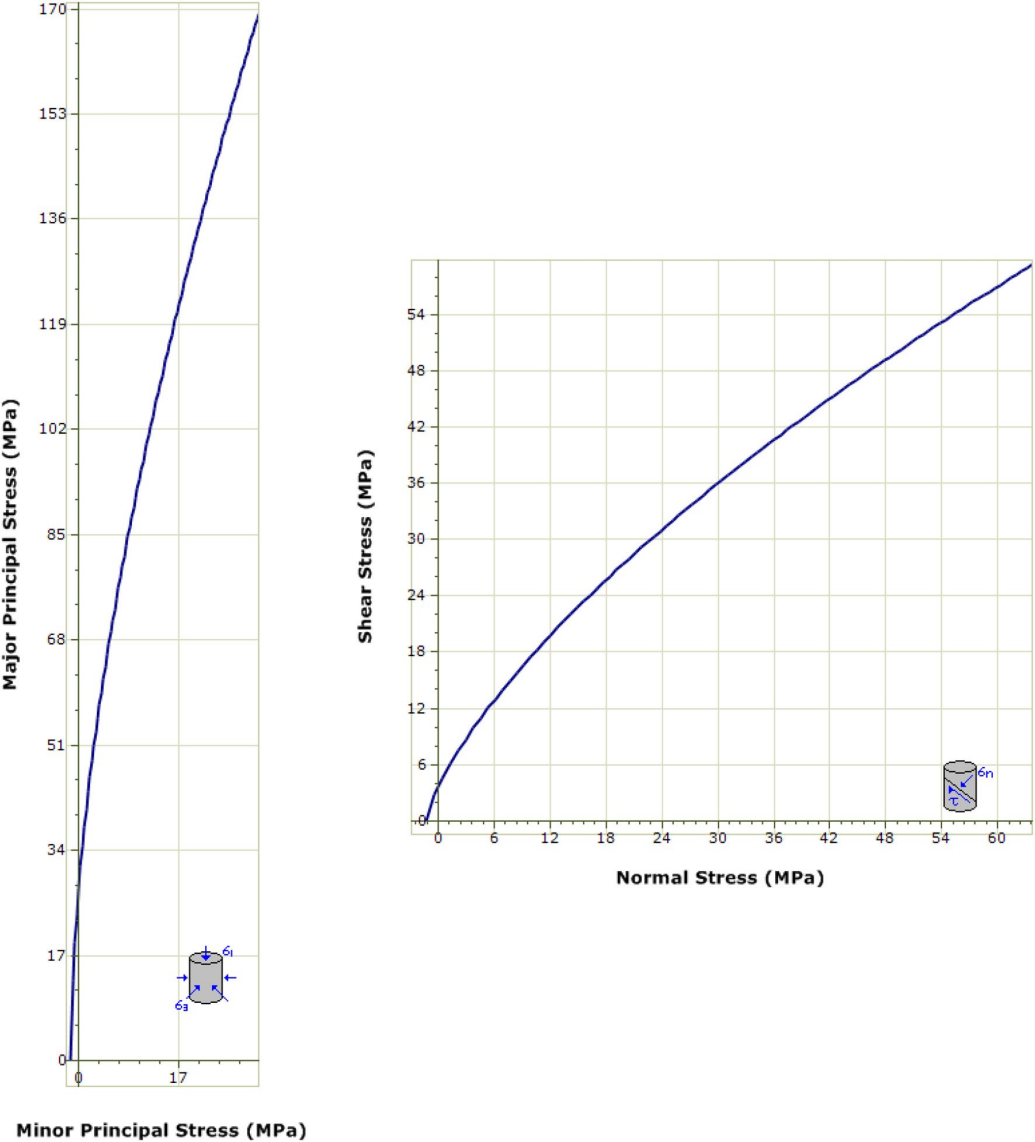
Hoek–Brown failure envelope and the mean values of geomechanical parameters

The *RocData* 5.0 software [49], was used to provide a deterministic estimation of the carbonatite-syenite rock mass parameters through the calculated mean values of the input parameters (Table 10). Similarly, Eqs. 1–6 were used by the software and the factor *D* was considered equal to 0 [50]. The failure envelopes of the Hoek–Brown criterion, traced for the calculated parameters, are presented in Fig. 12. Table 12 presented the average estimated values of strength and deformability parameters for the carbonatite-syenite rock mass.

**Table 12:** Deterministic calculation of the Hoek–Brown and Mohr–Coulomb failure constants for the carbonatite-syenite rock mass

*m*_*b*_	*s*	*a*	*σ*_*t*_ [MPa]	*σ*_*c*_ [MPa]	*σ*_*cm*_ [MPa]	*E*_*rm*_ [GPa]
5.137	0.056	0.501	1.284	27.872	41.039	49.194

## Discussion on the geomechanical characterization results

Studying the results of geological and geomechanical characterizations, emphasizes the necessity of adopting an accurate interpretation of data considering both lithological and geomechanical descriptions of a rock mass. In fact, lithological heterogeneity shouldn’t provide a misleading indication on the presence of geomechanical variety throughout that rock mass.

In this regard, studying the field and laboratory tests results of different test campaigns at the Niobec Mine, revealed that lithological heterogeneity rather than depth was responsible for the variation in geomechanical properties of the three main identified rock units. Studying the results proved that the largest variations in geomechanical parameters belong to the carbonatitic lithological units either with maximum separation from the syenitic lithological units or with intense alteration to chlorite. In fact, the resulted variability in strength and deformability parameters of intact samples was determined to be significantly defined by the degree of carbonatite alteration. Furthermore, geomechanical characterization of intact samples identified the carbonatitic units with a better quality than syenitic units. Determination of the Mohr–Coulomb and Hoek–Brown failure parameters for each constituent lithological unit and subsequently each rock unit (Tables 7 and 8), revealed that the strength of different carbonatitic lithological units was relatively constant except for the units either with intense alteration or with high presence of accessory minerals. In fact, according to the results of previously conducted triaxial tests, it was concluded that high concentrations of accessory minerals within the intact samples, presence of a contact between two different lithological units and presence of large quantities of altered syenite fragments to chlorite could result in reduction of rock strength compared to equivalent samples with more uniform lithological context and less concentration of micro cracks.

However, in geomechanical perspective, the observed difference between the calculated strength and deformability parameters of carbonatite and syenite units, wouldn’t be sufficient enough to justify the assumption of considering them as distinguished units within the intact rock; however, the carbonatite-syenite rock unit provided a quite reasonable approximation of geomechanical properties of the two aforementioned units. Besides, findings of local site characterization programs during the deposit exploitation phases, demonstrated that the nature of rock mass at the Niobec Mine is too complex to be discretized geomechanically and the captured irregularities in the quality of intact rock units are considered to occur in local scales [34, 35]. Therefore, assuming a homogeneous intact rock containing a random combination of syenite and carbonatite rock units could provide a valid geomechanical description of intact rock for the case of Niobec Mine.

In the rock mass scale, estimation of geomechanical parameters separately for syenite, carbonatite and carbonatite-syenite rock units also determined the quality of carbonatite rock mass to be slightly higher than syenite but fairly close to the quality of the carbonatite-syenite rock mass. Even the dispersions of the estimated strength and deformability parameters, were obtained to be very similar between carbonatite and carbonatite-syenite rock masses (Table 11). The obtained results of rock mass geomechanical characterization in this study, was based on estimating the GSI value using RMR_89_ conversion method. It should be noted that even though this conversion method has been conventionally used in many similar studies, it can be unreliable, particularly for poor quality rock masses and for rocks with lithological peculiarities that cannot be easily incorporated in the RMR calculations. Therefore, it is recommended to estimate the GSI directly and not from the RMR classification. Moreover, due to the lack of data, a same value of GSI had to be considered for all the three identified rock mass units which imposed a limit upon the accuracy of the obtained results through oversimplifying the calculation of rock mass geomechanical parameters for different units.

Ultimately, comparison between the results of deterministic and probabilistic estimation of geomechanical parameters for the particular case of carbonatite-syenite rock mass, proved the significant presence of variability associated to each parameter and inability of conventional deterministic approaches to address them entirely (Table 12). Conventional deterministic approaches cannot capture the variability of rock mass properties since they assign a single mean value to the parameters instead of defining them through probability distribution functions (which consider the mean and standard deviation values). This simplification not only ignores the inherent variable nature of rock mass, but also by assuming a better rock mass quality in terms of strength and stiffness produces unreliable and unclear results that could be misleading in subsequent rock mechanics analyses. It is hence recommended to incorporate uncertainties associated with the variability of rock mass parameters into consideration to ensure that the actual behavior of rock mass is properly reflected.

## Conclusion

The results of previously conducted geomechanical and geological field and laboratory tests at the Niobec Mine (Quebec, Canada) were combined to characterize the heterogeneous rock mass by considering the lithological and mechanical properties of identified lithological units’ constituents. The aim of this study was to find a reasonable agreement between the geomechanical parameters in relation to the extensive lithological variability for describing the rock mass properties.

The results of intact rock characterization indicated that the carbonatite-syenite rock unit could be considered as an appropriate representative lithology to define the rock mass geomechanical properties. Furthermore, estimated rock mass geomechanical parameters for syenitic, carbonatitic and carbonatitic-syenitic units also indicated that considering the carbonatite-syenite rock mass instead of trying to distinguish the syenite and the carbonatite as separate units provide a reasonable and reliable approximation of the geomechanical quality of rock mass at the Niobec Mine. Moreover, consideration of the carbonatite-syenite rock unit to represent the entire rock mass provides a good agreement between both the geomechanical and geological perspectives. Finally, the use probabilistic approaches instead of conventional deterministic methods in rock mass geomechanical characterization programs are highly recommended since a more realistic portray of the intrinsic nature of rock materials is depicted by considering the inherent variability associated to the geomechanical parameters.
